# Comparison of therapeutic effects of anterior decompression and posterior decompression on thoracolumbar spine fracture complicated with spinal nerve injury

**DOI:** 10.12669/pjms.312.6474

**Published:** 2015

**Authors:** Hongxun Cui, Jiayi Guo, Lei Yang, Yanxing Guo, Malong Guo

**Affiliations:** 1 Hongxun Cui, Orthopedic Hospital of Henan Province, Luoyang 471002, Henan Province, PR China; 2 Jiayi Guo, Orthopedic Hospital of Henan Province, Luoyang 471002, Henan Province, PR China; 3 Lei Yang, Orthopedic Hospital of Henan Province, Luoyang 471002, Henan Province, PR China; 4 Yanxing Guo, Orthopedic Hospital of Henan Province, Luoyang 471002, Henan Province, PR China; 5 Malong Guo, Orthopedic Hospital of Henan Province, Luoyang 471002, Henan Province, PR China

**Keywords:** Anterior decompression, Posterior decompression, Spinal nerve injury, Thoracolumbar spine fracture

## Abstract

**Objective:**

To compare the clinical therapeutic effects of anterior decompression and posterior decompression on thoracolumbar spine fracture (TSF) complicated with spinal nerve injury (SNI).

**Methods:**

A total of 120 patients with TSF and SNI were selected and divided into a treatment group and a control group that were then treated by anterior decompression and posterior decompression respectively. The preoperative and postoperative motor scores, tactile scores, heights of injured vertebral body and Cobb’s angles, as well as surgical times and intraoperative blood losses were recorded and compared.

**Results:**

Before surgeries, the motor score, tactile score, height of injured vertebral body and Cobb’s angle of the treatment group were similar to those of the control group (P>0.05). After surgeries, the values of the treatment group were significantly different from those of the control group (P<0.05). The two groups also had significantly different intraoperative blood losses and surgical times (P<0.05).

**Conclusion:**

Compared with posterior decompression, anterior decompression improved spinal cord function better and relived spinal cord compression more effectively with a more reasonable mechanics of internal fixation. Although this protocol caused more blood loss, the overall therapeutic effects were more satisfactory.

## INTRODUCTION

In clinical practice, patients with thoracolumbar spine fracture (TSF) are usually complicated with spinal cord injury, thus being accompanied by different extents of nerve disorders.^1^ If spinal cord compression is not relieved in time, the functions of injured nerves cannot recover and even worsen, so the daily life of patients is affected. It is of great significance to perform surgeries for the patients with TSF complicated with spinal nerve injury (SNI) to recover spinal cord function and to minimize the risk of disability. At present, such patients are treated either by anterior decompression or by posterior decompression, the clinical therapeutic effects of which were compared in this study.

## METHODS

### Ethics

The study protocol was approved by the Institutional Ethics Committee of Luoyang Orthopedic-Traumatological Hospital, and was in compliance with the Declaration of Helsinki. Written informed consent for abdominal paracentesis has been obtained from all patients.

### General information

A total of 120 patients with TSF and SNI enrolled in our hospital from January 2012 to February 2014 were selected and examined as kyphosis and spinal cord compression by X-ray and CR. They were then divided into a treatment group and a control group according to random number method (n=60). Control group: 35 males and 25 females; 22-58 years old, (46.0 ± 1.0) in average; causes of injury: 25 cases of falling injury, 20 cases of car accident injury and 15 cases of smashing injury; they were injured 0.6-19.5 h ago, (6.2 ± 1.3) h in average. Injured vertebral segments: 18 cases of T_11_ segment, 12 cases of T_12_ segment, 20 cases of L_1_ segment and 10 cases of L_2_ segment. Treatment group: 37 males and 23 females; 23-57 years old, (46.5 ± 1.0) in average; causes of injury: 30 cases of falling injury, 20 cases of car accident injury and 10 cases of smashing injury; they were injured 0.5-19.8 h ago, (6.5 ± 1.2) h in average. Injured vertebral segments: 21 cases of T_11_ segment, 12 cases of T_14_ segment, 18 cases of L_1_ segment and 7 cases of L_2_ segment. Clinical data of the two groups, such as causes of injury and ages, were similar (P>0.05).

### Diagnostic criteria

The patients were diagnosed according to the standards in “Practice of Orthopedics” and “Fractures and Joint Injuries”.^2^ They all had defined history of traumas, thoracolumbar fractures and limited motion. X-ray disclosed TSF, and CT disclosed spinal stenosis complicated with SNI.

### Inclusion criteria

Besides the criteria mentioned above, TSF only involved single segments, mainly distributed in the T_11_ and the L_2_ segments. Injury time <48 h; willing to participate in this study.

### Exclusion criteria

Some patients were excluded due to the following reasons: With multi-segmental fractures, pathological, old fractures or osteoporotic fractures; pregnant and lactating women who could not endure surgeries; with mental diseases; with juvenile spinal cord injury, TSF, ankylosing spondylitis complicated with fracture dislocation, or other severe diseases.

### Control group

This group was subjected to general anesthesia by tracheal intubation in the prone position, with the abdomen being suspended in the air.^3^ After accurate positioning with C-arm fluoroscopic imaging, a longitudinal incision was made in the middle of the back of thoracolumbar region, with the injured vertebra as the center. Afterwards, pedicle screws were implanted inside the upper and lower normal vertebral pedicles adjacent to the injured vertebra in an herringbone position.^4^ Then laminectomy was performed for the injured vertebra to explore compression to the ventral aspect of the spinal cord, and the fractures that intruded into the spinal canal were restored forward with an L-shaped push rod to relieve such compression. Subsequently, the nerve root and nerve root canal were explored again, and the patients with narrow nerve root canals were subjected to decompression. Finally, the position of internal fixation was confirmed by C-arm fluoroscopic imaging, and the incision was rinsed, hemostasized, and sutured layeredly. After the surgery, routine drain was placed, and antibiotics were given to prevent infections.

### Treatment group

This group was subjected to general anesthesia by tracheal intubation in the right lateral position, and the surgery was conducted by extra peritoneal access. For patients with fractures at the L_1_ segment, a curved incision was made along the 12th rib downwards from the outer edge of the paraspinal muscles to front of the upper left iliac crest.^5^ Then the superficial and deep fasciae were incised layeredly, and the muscles were incised before separating and resecting the 12th rib, aiming to find intercostal nerves and blood vessels that were thereafter ligated and severed.^6^ In addition, intraspinal fractured bones and intervertebral disc tissues were eliminated. Before decompression, screws were implanted into the upper and lower normal vertebrae adjacent to the injured vertebra. Eventually, the position of internal fixation was confirmed by C-arm fluoroscopic imaging, and the incision was rinsed, hemostasized, and sutured layeredly. After the surgery, routine drain was placed, and antibiotics were given to prevent infections.

### Observation indices

The surgical times, intraoperative blood losses, as well as preoperative and postoperative tactile scores, Cobb’s angles, motor scores and heights of injured vertebral body were recorded. Surgical time: The time from incision to suture. Intraoperative blood loss: Total volume of blood absorbed by dressings + blood in suction pump.^4^ Standards for evaluating tactile and motor scores: The scores were obtained according to the ASIA classification standards.^7^

### Statistical analysis

All data were analyzed by SPSS19.0. Motor score, Cobb’s angle, height of injured vertebral body, surgical time and intraoperative blood loss were expressed as mean ± standard deviation (x̄ ± s). The data were compared by t test. P<0.05 was considered statistically significant.

## RESULTS

### Clinical outcomes

Before surgeries, the motor score, tactile score, height of injured vertebral body and Cobb’s angle of the treatment group were (39.2 ± 17.2) point, (44.9 ± 16.1) point, (1.4 ± 0.6) cm and (21 ± 4)° respectively, which were similar to those of the control group [(39.9 ± 17.4) point, (45.3 ± 15.8) point, (1.5 ± 0.6) cm and (20 ± 5)°] (P>0.05). After surgeries, the motor score, tactile score, height of injured vertebral body and Cobb’s angle of the treatment group [(69.9 ± 23.1) point, (72.5 ± 15.6) point, (4.1 ± 0.4) cm and (43 ± 5)°] were significantly different from those of the control group [(52.8 ± 24.8) point, (61.2 ± 15.9) point, (2.4 ± 0.8) cm and (29 ± 6)°] (P<0.05) (Table-I).

**Table-I:** Clinical outcomes (x̄± s).

Group	Time	Motor score (point)	Tactile score (point)	Height of injured vertebral body (cm)	Cobb’s angle (°)
Control group (n=60)	Before	39.9±17.4	45.3±15.8	1.5±0.6	20±5
	After	52.8±24.8	61.2±15.9	2.4±0.8	29±6
t		-3.852	-7.452	-4.029	-3.055
P		<0.05	<0.05	<0.05	<0.05
Treatment group (n=60)	Before	39.2±17.2	44.9±16.1	1.4±0.6	21±4
	After	69.9±23.1	72.5±15.6	4.1±0.4	43±5
t		-5.893	-4.785	-4.318	-5.208
P		<0.05	<0.05	<0.05	<0.05
ta		0.852	0.694	0.686	0.765
P		>0.05	>0.05	>0.05	>0.05
tb		3.659	3.211	1.330	2.678
P		<0.05	<0.05	>0.05	<0.05

### Intraoperative blood loss and surgical time

The two groups had significantly different intraoperative blood losses and surgical times (P<0.05) (Table-II).

**Table-II:** Intraoperative blood loss and surgical time (±s)

Group	Intraoperative blood loss (ml)	Surgical time (min)
Treatment group (n=60)	498.6±24.7	217.6±19.8
Control group (n=60)	275.6±18.5	154.6±16.3
t	4.316	3.687
P	<0.05	<0.05

### X-ray images

**Fig.1:**
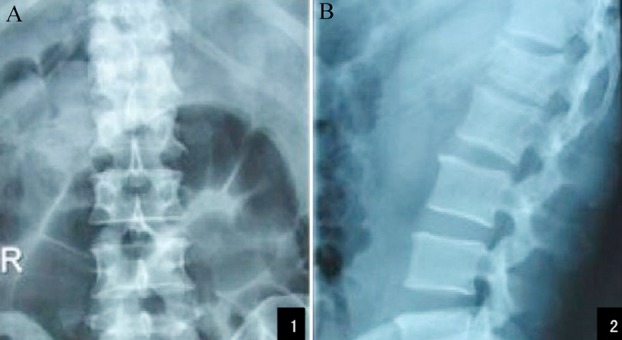
Preoperative anteroposterior (A) and lateral (B) X-ray images of L_1_ vertebra.

**Fig.2:**
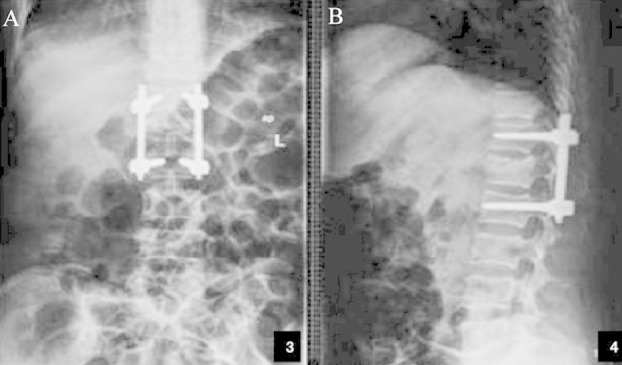
Postoperative anteroposterior (A) and lateral (B) X-ray images of L_1_ vertebra in the control group.

**Fig.3:**
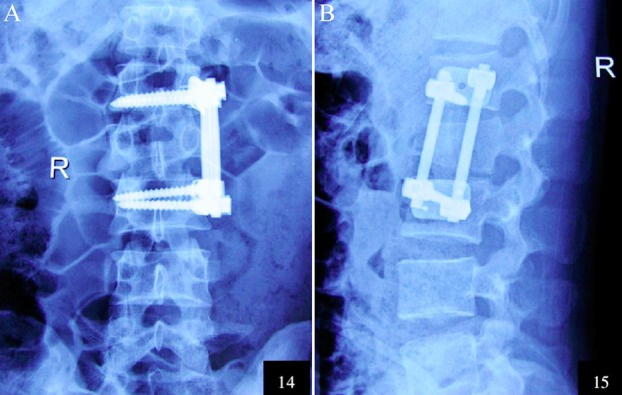
Postoperative anteroposterior (A) and lateral (B) X-ray images of L_1_ vertebra in the treatment group.

The X-ray images before surgeries as well as those of the control group and the treatment group after surgeries are shown in Fig. 1-3 respectively.

## DISCUSSION

The thoracolumbar spine, which mainly refers the T_11_-L_2_ spinal segments of human body, is located at the intersection of two physiological spinal curvatures.^8^ Without being protected by ribs or the thoracic cage, these spinal segments cannot resist intense injuries, so they are prone to burst fractures.^9^ Moreover, since the thoracolumbar spine is generally responsible for moving and fixing the lumbar vertebra, it may easily fracture due to concentrated stress, accompanied by spinal cord injury. As a result, the spine loses the load-carrying capacity, and patients, caused by nerve compression, suffer from different extents of movement disorders and lower limb sensory disorders, and even paraplegia. Furthermore, the thoracolumbar spine can only move within a small range, at which the thoracic facet joint surface shifts to the lumbar facet joint surface. Meanwhile, it is the turning point of posterior thoracic convexity and anterior lumbar convexity. Containing spinal cord and cauda equina, the thoracolumbar spine may partly recover in spinal nerve roots.

Posterior decompression is advantageous in mild intraoperative blood loss and quick postoperative recovery.^10^ Lin et al.^11^ reported that anterior and posterior decompressions were sufficient for surgically treating thoracolumbar burst fractures, and that posterior surgery allowed shorter operational time, less intraoperative blood loss and complications and better pulmonary function. Nevertheless, this method may fail upon anterior spinal compression that hinders effective removal of compressed tissues and fractures. Upon severe violent injuries, the patients with thoracolumbar fractures are mainly manifested as considerable height losses of anterior and middle vertebral bodies as well as intervertebral instability, thus reducing the bearing and support forces of the anterior column. Since the spinal cord is injured by being compressed with the intervertebral disc tissues in front of the spinal dura mater and fractured bones, anterior decompression is more suitable for alleviating or eliminating spinal cord compression under direct vision. In the meantime, this method can prevent posterior decompression-induced traction injuries and thus indirectly protect the spinal cord.^12^ Sudo et al.^13^ reported that anterior decompression was more suitable for osteoporotic vertebral collapse because anterior elements, particularly those at the thoracolumbar junction, predominantly controlled load bearing. Suzuki et al.^14^ found that an anterior or combined anteroposterior approach was required for surgical decompression to repair severely unstable lumbar burst fractures, with which a posterior approach was combined to improve the therapeutic effects.

In this study, the preoperative motor score, tactile score, height of injured vertebral body and Cobb’s angle of the treatment group were (39.2 ± 17.2) point, (44.9 ± 16.1) point, (1.4 ± 0.6) cm and (21 ± 4)° respectively, which were similar to those of the control group [(39.9 ± 17.4) point, (45.3 ± 15.8) point, (1.5 ± 0.6) cm and (20 ± 5)°] (P>0.05). After surgeries, however, the values of the treatment group were significantly better than those of the control group (P<0.05), despite more intraoperative blood loss and longer surgical time.

In summary, compared with posterior decompression, anterior decompression improved spinal cord function better and relived spinal cord compression more effectively with a more reasonable mechanics of internal fixation. Regardless, this complicated surgery cost more time, during which the patients also bled more. Meanwhile, they took longer time to fully recover owing to larger traumas. Particular attention should be paid to possible risks of hemorrhage and hemopneumothorax. Hence, it is crucial to relieve patients’ suffering by elaborate preparation and surgical time shortening.
